# Supplementary material for: Regional differences in agricultural and socioeconomic factors associated with farmer household dietary diversity in India
Source: PLoS One. 2020 Apr 16;15(4):e0231107. doi: 10.1371/journal.pone.0231107 (PMC7161949; doi:10.1371/journal.pone.0231107)
Supplement: S1 Table — (DOCX) [file pone.0231107.s001.docx]

Table S1: Calculation of a district-wise Farming Intensity Index (FII) for Gujarat

| **Serial Number** | **District** | **Crop Diversity (2013-14)** | | | **Livestock/person (2012)** | | | **Poultry/person (2012)** | | | **Rural Literacy (2011)** | | | **Income: Total cropped as % of total land area (2012-13)** | | | **Weighted Average (A+B+C+D+E)** | **Overall Ranking** |
| --- | --- | --- | --- | --- | --- | --- | --- | --- | --- | --- | --- | --- | --- | --- | --- | --- | --- | --- |
|  |  | **CDI** | **(X-mean)/ SD** | **Rank** | **Livestock/ Person** | **(X-mean) /SD** | **Rank** | **Poultry /person** | **(X-mean) /SD** | **Rank** | **Rural Literacy (%)** | **(X-mean) /SD** | **Rank** | **Total cropped area to total area (%)** | **(X-mean)/ SD** | **Rank** |  |  |
|  |  |  | **A** |  |  | **B** |  |  | **C** |  |  | **D** |  |  | **E** |  |  |  |
| 1 | Ahmedabad | 0.78 | 0.29 | 15 | 0.11 | -1.85 | 26 | 0.05129 | -0.59 | 19 | 71.05 | -0.18 | 14 | 73.26 | 0.67 | 7 | -0.17 | 17 |
| 2 | Amreli | 0.45 | -2.02 | 26 | 0.72 | 0.99 | 6 | 0.0066 | -0.69 | 25 | 71.77 | -0.07 | 13 | 54.87 | -0.10 | 14 | -0.98 | 23 |
| 3 | Anand | 0.83 | 0.66 | 11 | 0.37 | -0.63 | 20 | 2.14312 | 4.11 | 1 | 82.68 | 1.62 | 1 | 102.28 | 1.88 | 1 | 1.20 | 1 |
| **4** | **Banas Kantha** | **0.90** | **1.17** | **1** | **0.83** | **1.51** | **4** | **0.08909** | **-0.51** | **17** | **62.91** | **-1.44** | **25** | **84.50** | **1.14** | **4** | **0.71** | **2** |
| 5 | Bharuch | 0.77 | 0.25 | 16 | 0.26 | -1.17 | 24 | 0.18955 | -0.28 | 14 | 77.99 | 0.89 | 6 | 43.79 | -0.57 | 18 | -0.06 | 15 |
| **6** | **Bhavnagar** | **0.48** | **-1.78** | **25** | **0.42** | **-0.43** | **18** | **0.01458** | **-0.67** | **24** | **70.7** | **-0.24** | **15** | **45.10** | **-0.51** | **17** | **-1.13** | **25** |
| 7 | Dahod | 0.82 | 0.58 | 12 | 0.84 | 1.59 | 3 | 0.39303 | 0.18 | 8 | 56.36 | -2.46 | 26 | 95.77 | 1.61 | 2 | 0.47 | 7 |
| 8 | Dangs | 0.84 | 0.75 | 8 | 0.60 | 0.42 | 8 | 0.84541 | 1.19 | 2 | 73.42 | 0.18 | 10 | 26.27 | -1.30 | 25 | 0.50 | 6 |
| 9 | Gandhinagar | 0.84 | 0.73 | 9 | 0.42 | -0.41 | 17 | 0.09556 | -0.49 | 15 | 81.57 | 1.45 | 4 | 65.61 | 0.35 | 9 | 0.41 | 10 |
| 10 | Godhara | 0.81 | 0.51 | 14 | 0.85 | 1.60 | 2 | 0.22545 | -0.20 | 13 | 68.36 | -0.60 | 20 | 26.73 | -1.28 | 24 | 0.27 | 13 |
| 11 | Jamnagar | 0.63 | -0.73 | 19 | 0.50 | -0.05 | 11 | 0.01667 | -0.67 | 22 | 69.03 | -0.50 | 19 | 52.93 | -0.18 | 15 | -0.54 | 20 |
| 12 | Junagadh | 0.54 | -1.36 | 23 | 0.45 | -0.27 | 15 | 0.06453 | -0.56 | 18 | 72.61 | 0.06 | 11 | 9.69 | -2.00 | 26 | -1.00 | 24 |
| 13 | Kachchh | 0.86 | 0.86 | 5 | 0.93 | 1.99 | 1 | 0.0239 | -0.65 | 21 | 64.92 | -1.13 | 24 | 94.46 | 1.56 | 3 | 0.67 | 5 |
| 14 | Kheda | 0.86 | 0.87 | 4 | 0.54 | 0.14 | 10 | 0.24436 | -0.16 | 11 | 81.42 | 1.42 | 5 | 84.41 | 1.14 | 5 | 0.69 | 4 |
| 15 | Mahesana | 0.87 | 0.93 | 3 | 0.47 | -0.20 | 13 | 0.09435 | -0.49 | 16 | 81.97 | 1.51 | 2 | 41.57 | -0.66 | 19 | 0.45 | 8 |
| 16 | Narmada | 0.84 | 0.78 | 7 | 0.57 | 0.31 | 9 | 0.27444 | -0.09 | 9 | 70.46 | -0.27 | 16 | 39.58 | -0.74 | 20 | 0.32 | 12 |
| 17 | Navsari | 0.63 | -0.76 | 20 | 0.33 | -0.83 | 22 | 0.6573 | 0.77 | 3 | 81.65 | 1.46 | 3 | 63.05 | 0.24 | 10 | -0.22 | 18 |
| 18 | Patan | 0.87 | 0.97 | 2 | 0.47 | -0.19 | 12 | 0.02456 | -0.65 | 20 | 69.33 | -0.45 | 18 | 59.29 | 0.08 | 13 | 0.32 | 11 |
| 19 | Porbandar | 0.48 | -1.78 | 24 | 0.46 | -0.20 | 14 | 0.01537 | -0.67 | 23 | 69.39 | -0.44 | 17 | 33.85 | -0.98 | 22 | -1.16 | 26 |
| 20 | Rajkot | 0.60 | -0.93 | 21 | 0.36 | -0.67 | 21 | 0.25259 | -0.14 | 10 | 74.65 | 0.38 | 8 | 62.64 | 0.22 | 11 | -0.53 | 19 |
| 21 | Sabarkantha | 0.85 | 0.80 | 6 | 0.77 | 1.23 | 5 | 0.2417 | -0.16 | 12 | 74.19 | 0.30 | 9 | 81.85 | 1.03 | 6 | 0.69 | 3 |
| 22 | Surat | 0.70 | -0.24 | 17 | 0.43 | -0.36 | 16 | 0.52498 | 0.47 | 6 | 76.92 | 0.73 | 7 | 38.12 | -0.80 | 21 | -0.11 | 16 |
| 23 | Surendranagar | 0.64 | -0.68 | 18 | 0.20 | -1.44 | 25 | 0.00132 | -0.70 | 26 | 67.95 | -0.66 | 21 | 62.24 | 0.21 | 12 | -0.70 | 21 |
| 24 | Tapi | 0.84 | 0.73 | 10 | 0.62 | 0.54 | 7 | 0.64682 | 0.75 | 4 | 66.47 | -0.89 | 23 | 45.17 | -0.51 | 16 | 0.42 | 9 |
| **25** | **Vadodara** | **0.81** | **0.52** | **13** | **0.40** | **-0.49** | **19** | **0.39514** | **0.18** | **7** | **67.84** | **-0.68** | **22** | **69.73** | **0.52** | **8** | **0.20** | **14** |
| 26 | Valsad | 0.58 | -1.10 | 22 | 0.27 | -1.13 | 23 | 0.63435 | 0.72 | 5 | 72.32 | 0.01 | 12 | 33.58 | -0.99 | 23 | -0.71 | 22 |
|  | **X = mean** | **0.73** | **0.00** |  | **0.51** | **0.00** |  | **0.31** | **0.00** |  | **72.23** | **0.00** |  | **57.32** | **0.00** |  | **0.00** |  |
|  | **SD** | **0.14** |  |  | **0.21** |  |  | **0.45** |  |  | **6.46** |  |  | **23.87** |  |  |  |  |

The bold ones are the selected districts for survey

Source: Table from Singh et al. 2020
