# Supplementary material for: Regional differences in agricultural and socioeconomic factors associated with farmer household dietary diversity in India
Source: PLoS One. 2020 Apr 16;15(4):e0231107. doi: 10.1371/journal.pone.0231107 (PMC7161949; doi:10.1371/journal.pone.0231107)
Supplement: S2 Table — (DOCX) [file pone.0231107.s002.docx]

Table S2: Calculation of a district-wise Farming Intensity Index (FII) for Haryana

| **Serial Number** | **District** | **Crop Diversity (2013-14)** | | | **Livestock/person (2012)** | | | **Poultry/person (2012)** | | | **Rural Literacy (2011)** | | | **Income: Total cropped as % of total land area (2013-14)** | | | **Weighted Average (A+B+C+D+E)** | **Overall Ranking** |
| --- | --- | --- | --- | --- | --- | --- | --- | --- | --- | --- | --- | --- | --- | --- | --- | --- | --- | --- |
|  |  | **CDI** | **(X-mean)**  **/SD** | **Rank** | **Livestock /person** | **(X-mean) / SD** | **Rank** | **Poultry /person** | **(X-mean) /SD** | **Rank** | **Rural Literacy (%)** | **(X-mean) /SD** | **Rank** | **Total cropped area to total area (%)** | **(X-mean) /SD** | **Rank** |  |  |
|  |  |  | **A** |  |  | **B** |  |  |  |  |  | **C** |  |  | **E** |  |  |  |
| 1 | Hisar | 0.78 | 1.25 | 2 | 0.44 | 0.70 | 6 | 2.07 | 0.07 | 6 | 68.74 | -0.51 | 16 | 166.21 | 0.99 | 4 | 0.79 | 2 |
| 2 | Fatehabad | 0.70 | 0.08 | 11 | 0.50 | 1.23 | 4 | 0.31 | -0.62 | 17 | 65.52 | -1.00 | 19 | 85.02 | -1.40 | 19 | -0.11 | 10 |
| 3 | Sirsa | 0.73 | 0.55 | 8 | 0.52 | 1.37 | 2 | 0.43 | -0.57 | 15 | 65.41 | -1.02 | 20 | 168.11 | 1.05 | 3 | 0.40 | 6 |
| **4** | **Bhiwani** | **0.82** | **1.95** | **1** | **0.48** | **1.08** | **5** | **1.73** | **-0.06** | **8** | **73.67** | **0.24** | **10** | **154.88** | **0.66** | **6** | **1.22** | **1** |
| 5 | Rohtak | 0.75 | 0.80 | 6 | 0.34 | -0.10 | 11 | 0.66 | -0.48 | 14 | 76.81 | 0.72 | 7 | 132.38 | -0.01 | 12 | 0.38 | 7 |
| 6 | Jhajjar | 0.76 | 0.98 | 3 | 0.37 | 0.14 | 8 | 0.23 | -0.65 | 18 | 79.39 | 1.11 | 3 | 128.14 | -0.13 | 13 | 0.51 | 5 |
| 7 | Sonepat | 0.65 | -0.78 | 16 | 0.34 | -0.15 | 12 | 1.08 | -0.32 | 10 | 76.93 | 0.74 | 5 | 136.66 | 0.12 | 11 | -0.38 | 16 |
| 8 | Gurgaon | 0.72 | 0.30 | 10 | 0.17 | -1.60 | 20 | 0.33 | -0.61 | 16 | 80.08 | 1.22 | 1 | 87.44 | -1.33 | 18 | -0.19 | 11 |
| **9** | **Mewat** | **0.74** | **0.67** | **7** | **0.29** | **-0.59** | **17** | **0.04** | **-0.72** | **11** | **51.99** | **-3.06** | **21** | **118.12** | **-0.43** | **16** | **-0.21** | **12** |
| 10 | Faridabad | 0.68 | -0.33 | 14 | 0.10 | -2.18 | 21 | 0.02 | -0.73 | 20 | 73.18 | 0.16 | 11 | 85.02 | -1.40 | 20 | -0.72 | 21 |
| 11 | Palwal | 0.68 | -0.34 | 15 | 0.34 | -0.15 | 13 | 0.01 | -0.74 | 21 | 66.72 | -0.82 | 17 | 142.75 | 0.30 | 10 | -0.35 | 15 |
| 12 | Panipat | 0.62 | -1.23 | 19 | 0.27 | -0.76 | 18 | 1.90 | 0.00 | 7 | 72.50 | 0.06 | 13 | 148.26 | 0.46 | 9 | -0.68 | 19 |
| **13** | **Karnal** | **0.59** | **-1.81** | **21** | **0.37** | **0.12** | **9** | **4.36** | **0.97** | **3** | **71.37** | **-0.11** | **14** | **150.79** | **0.54** | **8** | **-0.70** | **20** |
| 14 | Kurukshetra | 0.63 | -1.16 | 18 | 0.36 | -0.01 | 10 | 3.76 | 0.73 | 4 | 73.11 | 0.15 | 12 | 177.78 | 1.33 | 1 | -0.32 | 14 |
| 15 | Kaithal | 0.60 | -1.50 | 20 | 0.52 | 1.44 | 1 | 1.48 | -0.16 | 9 | 66.67 | -0.83 | 18 | 164.01 | 0.93 | 5 | -0.55 | 17 |
| 16 | Ambala | 0.63 | -1.15 | 17 | 0.30 | -0.51 | 16 | 2.26 | 0.15 | 5 | 77.13 | 0.77 | 4 | 126.43 | -0.18 | 14 | -0.57 | 18 |
| 17 | Panchkula | 0.75 | 0.81 | 5 | 0.20 | -1.36 | 19 | 11.04 | 3.59 | 1 | 75.64 | 0.54 | 8 | 49.00 | -2.47 | 21 | 0.55 | 4 |
| 18 | Yamunanagar | 0.69 | -0.15 | 12 | 0.30 | -0.45 | 15 | 0.83 | -0.41 | 12 | 73.80 | 0.26 | 9 | 117.65 | -0.44 | 17 | -0.22 | 13 |
| 19 | Jind | 0.69 | -0.18 | 13 | 0.51 | 1.31 | 3 | 5.20 | 1.30 | 2 | 68.85 | -0.50 | 15 | 174.69 | 1.24 | 2 | 0.38 | 8 |
| 20 | Mohendragarh | 0.76 | 0.88 | 4 | 0.44 | 0.70 | 7 | 1.03 | -0.34 | 19 | 76.88 | 0.73 | 6 | 151.66 | 0.56 | 7 | 0.63 | 3 |
| 21 | Rewari | 0.72 | 0.35 | 9 | 0.33 | -0.23 | 14 | 0.82 | -0.42 | 13 | 79.69 | 1.16 | 2 | 119.82 | -0.38 | 15 | 0.16 | 9 |
|  | **X=mean** | **0.70** | **0.00** |  | **0.36** | **0.00** |  | **1.89** | **0.00** |  | **72.10** | **0.00** |  | **132.61** |  |  | **0.00** |  |
|  | **SD** | **0.06** |  |  | **0.12** |  |  | **2.55** |  |  | **6.56** |  |  | **33.88** |  |  |  |  |

The bold ones are the selected districts for survey

Source: Table from Singh et al. 2020
