# Supplementary material for: Regional differences in agricultural and socioeconomic factors associated with farmer household dietary diversity in India
Source: PLoS One. 2020 Apr 16;15(4):e0231107. doi: 10.1371/journal.pone.0231107 (PMC7161949; doi:10.1371/journal.pone.0231107)
Supplement: S3 Table — (DOCX) [file pone.0231107.s003.docx]

Table S3: Calculation of a block-wise Farming Intensity Index (FII) for districts in Gujarat

| **Serial Number** | **Block** | **Crop Diversity (2015-16)** | | | **Rural Literacy (2011)** | | | **Total cropped as % of total land area (2015-16)** | | | **Weighted Average (A+B+C)** | **Overall Ranking** |
| --- | --- | --- | --- | --- | --- | --- | --- | --- | --- | --- | --- | --- |
|  |  | **CDI** | **(X-mean)/ SD** | **Rank** | **Rural Literacy (%)** | **(X-mean)/ SD** | **Rank** | **Total cropped area to total area (%)** | **(X-mean)/ SD** | **Rank** |  |  |
| **BANAS KANTHA** | |  | **A** |  |  | **B** |  |  | **C** |  |  |  |
| **1** | **Palanpur** | **0.88** | **1.01** | **2** | **74.95** | **1.88** | **2** | **128.55** | **1.01** | **4** | **1.18** | **1** |
| 2 | Amirgadh | 0.82 | -0.76 | 11 | 50.85 | -1.56 | 14 | 39.05 | -1.41 | 13 | -1.05 | 12 |
| 3 | Danta | 0.78 | -1.83 | 14 | 62.05 | 0.04 | 5 | 36.53 | -1.47 | 14 | -1.38 | 14 |
| 4 | Vadgam | 0.81 | -0.79 | 12 | 77.85 | 2.29 | 1 | 72.12 | -0.51 | 10 | -0.12 | 9 |
| 5 | Disa | 0.85 | 0.16 | 8 | 61.59 | -0.03 | 6 | 137.09 | 1.24 | 1 | 0.34 | 7 |
| 6 | Dhanera | 0.83 | -0.21 | 9 | 56.42 | -0.76 | 12 | 84.80 | -0.17 | 9 | -0.32 | 10 |
| 7 | Dantiwada | 0.87 | 0.90 | 3 | 62.68 | 0.13 | 4 | 103.71 | 0.34 | 7 | 0.63 | 4 |
| 8 | Kankrej | 0.88 | 1.09 | 1 | 59.63 | -0.31 | 8 | 129.82 | 1.04 | 2 | 0.80 | 2 |
| 9 | Diyodar | 0.86 | 0.54 | 7 | 63.55 | 0.25 | 3 | 108.53 | 0.47 | 6 | 0.47 | 6 |
| 10 | Bhabhar | 0.87 | 0.72 | 6 | 56.11 | -0.81 | 13 | 96.39 | 0.14 | 8 | 0.30 | 8 |
| 11 | Vav | 0.82 | -0.61 | 10 | 59.40 | -0.34 | 9 | 46.84 | -1.20 | 11 | -0.67 | 11 |
| 12 | Tharad | 0.87 | 0.75 | 5 | 58.91 | -0.41 | 11 | 119.26 | 0.76 | 5 | 0.52 | 5 |
| 13 | Lakhani | 0.87 | 0.80 | 4 | 61.59 | -0.03 | 7 | 128.62 | 1.01 | 3 | 0.67 | 3 |
| **14** | **Suigam** | **0.78** | **-1.73** | **13** | **59.40** | **-0.34** | **10** | **45.24** | **-1.24** | **12** | **-1.35** | **13** |
|  | **X=mean** | **0.84** | **0.00** |  | **61.78** | **0.00** |  | **91.18** | **0.00** |  |  |  |
|  | **SD** | **0.04** |  |  | **7.02** |  |  | **37.06** |  |  |  |  |
| **BHAVNAGAR** | |  |  |  |  |  |  |  |  |  |  |  |
| 1 | Vallabhipur | 0.48 | -0.93 | 9 | 72.55 | 0.22 | 5 | 76.31 | 0.06 | 6 | -0.50 | 9 |
| **2** | **Umrala** | **0.27** | **-2.15** | **10** | **72.40** | **0.18** | **6** | **79.23** | **0.23** | **4** | **-1.21** | **10** |
| 3 | Bhavnagar | 0.75 | 0.56 | 5 | 74.23 | 0.66 | 3 | 36.72 | -2.17 | 10 | 0.03 | 6 |
| **4** | **Ghogha** | **0.82** | **0.93** | **1** | **77.73** | **1.57** | **1** | **73.16** | **-0.11** | **7** | **0.85** | **1** |
| 5 | Sihor | 0.62 | -0.17 | 7 | 72.26 | 0.15 | 7 | 69.19 | -0.34 | 8 | -0.14 | 7 |
| 6 | Gariadhar | 0.52 | -0.75 | 8 | 74.69 | 0.78 | 2 | 77.50 | 0.13 | 5 | -0.27 | 8 |
| 7 | Palitana | 0.71 | 0.33 | 6 | 72.84 | 0.30 | 4 | 62.79 | -0.70 | 9 | 0.12 | 5 |
| 8 | Talaja | 0.80 | 0.84 | 2 | 68.60 | -0.81 | 8 | 102.01 | 1.51 | 1 | 0.65 | 2 |
| 9 | Mahuva | 0.76 | 0.64 | 4 | 65.83 | -1.53 | 9 | 95.08 | 1.12 | 2 | 0.30 | 3 |
| 10 | Jesar | 0.78 | 0.72 | 3 | 65.83 | -1.53 | 10 | 79.79 | 0.26 | 3 | 0.18 | 4 |
|  | **X=mean** | **0.65** | **0.00** |  | **71.70** | **0.00** |  | **75.18** | **0.00** |  |  |  |
|  | **SD** | **0.18** |  |  | **3.84** |  |  | **17.74** |  |  |  |  |
| **VADODARA** | |  |  |  |  |  |  |  |  |  |  |  |
| 1 | Savli | 0.86 | 0.63 | 1 | 78.29 | -0.57 | 5 | 53.83 | -0.54 | 6 | 0.15 | 3 |
| **2** | **Vadodara** | **0.85** | **0.55** | **3** | **84.77** | **2.26** | **1** | **63.07** | **0.46** | **3** | **0.87** | **1** |
| 3 | Vaghodia | 0.77 | 0.16 | 6 | 80.76 | 0.51 | 2 | 46.46 | -1.33 | 8 | -0.07 | 6 |
| 4 | Dabhoi | 0.82 | 0.40 | 5 | 78.00 | -0.70 | 8 | 54.67 | -0.45 | 5 | 0.01 | 5 |
| 5 | Padra | 0.82 | 0.40 | 4 | 79.49 | -0.05 | 3 | 76.71 | 1.93 | 1 | 0.62 | 2 |
| 6 | Karjan | 0.68 | -0.37 | 7 | 79.12 | -0.21 | 4 | 64.43 | 0.60 | 2 | -0.14 | 7 |
| **7** | **Sinor** | **0.31** | **-2.34** | **8** | **78.08** | **-0.66** | **7** | **58.85** | **0.00** | **4** | **-1.54** | **8** |
| 8 | Desar | 0.85 | 0.58 | 2 | 78.29 | -0.57 | 6 | 52.61 | -0.67 | 7 | 0.10 | 4 |
|  | **X=mean** | **0.74** | **0.00** |  | **79.60** | **0.00** |  | **58.83** | **0.00** |  |  |  |
|  | **SD** | **0.19** |  |  | **2.29** |  |  | **9.28** |  |  |  |  |

The bold ones are the selected blcks for survey

Source: Table from Singh et al. 2020
