# Supplementary material for: Regional differences in agricultural and socioeconomic factors associated with farmer household dietary diversity in India
Source: PLoS One. 2020 Apr 16;15(4):e0231107. doi: 10.1371/journal.pone.0231107 (PMC7161949; doi:10.1371/journal.pone.0231107)
Supplement: S4 Table — (DOCX) [file pone.0231107.s004.docx]

Table S4: Calculation of a block-wise Farming Intensity Index (FII) for districts in Haryana

| **Serial Number** | **Block** | **Crop Diversity (2013-14)** | | | **Rural Literacy (2011)** | | | **Total cropped as % of total land area (2013-14)** | | | **Weighted Average (A+B+C)** | **Overall Ranking** |
| --- | --- | --- | --- | --- | --- | --- | --- | --- | --- | --- | --- | --- |
|  |  | **CDI** | **(X-mean)/ SD** | **Rank** | **Rural Literacy (%)** | **(X-mean)/ SD** | **Rank** | **Total cropped area to total area (%)** | **(X-mean)/ SD** | **Rank** |  |  |
| **Bhiwani** | |  | **A** |  |  | **B** |  |  | **C** |  |  |  |
| 1 | Loharu | 0.75 | 0.12 | 5 | 68.74 | 1.66 | 1 | 123.85 | -0.04 | 5 | 0.40 | 4 |
| **2** | **Bhiwani** | **0.79** | **1.05** | **1** | **64.12** | **0.02** | **5** | **131.50** | **0.68** | **3** | **0.77** | **2** |
| 3 | Tosham | 0.78 | 0.90 | 3 | 60.89 | -1.13 | 9 | 120.08 | -0.39 | 6 | 0.23 | 5 |
| 4 | Bhiwani khera | 0.72 | -0.43 | 8 | 62.90 | -0.42 | 7 | 146.70 | 2.09 | 1 | 0.08 | 6 |
| 5 | Dadri-I -Bond kalan | 0.79 | 1.02 | 2 | 67.40 | 1.19 | 2 | 132.79 | 0.80 | 2 | 1.01 | 1 |
| 6 | Dadri-II | 0.76 | 0.40 | 4 | 66.25 | 0.78 | 3 | 127.46 | 0.30 | 4 | 0.45 | 3 |
| 7 | Badhara | 0.74 | 0.03 | 6 | 64.85 | 0.28 | 4 | 114.34 | -0.92 | 9 | -0.11 | 7 |
| 8 | Siwani | 0.74 | -0.09 | 7 | 59.81 | -1.52 | 10 | 110.20 | -1.31 | 10 | -0.62 | 8 |
| 9 | Kairu | 0.71 | -0.74 | 9 | 63.08 | -0.35 | 6 | 118.53 | -0.53 | 7 | -0.62 | 9 |
| **10** | **Behal** | **0.64** | **-2.26** | **10** | **62.64** | **-0.51** | **8** | **117.11** | **-0.67** | **8** | **-1.59** | **10** |
|  | **X=mean** | **0.74** | **0.00** |  | **64.07** | **0.00** |  | **124.26** | **0.00** |  |  |  |
|  | **SD** | **0.04** |  |  | **2.81** |  |  | **10.73** |  |  |  |  |
| **Karnal** | |  |  |  |  |  |  |  |  |  |  |  |
| 1 | Karnal | 0.52 | -0.46 | 4 | 61.90 | 0.04 | 3 | 172.40 | 0.97 | 2 | -0.07 | 3 |
| **2** | **Nissing** | **0.51** | **-0.86** | **6** | **61.48** | **-0.16** | **4** | **139.51** | **-0.27** | **4** | **-0.60** | **6** |
| **3** | **Indri** | **0.59** | **1.71** | **1** | **64.78** | **1.36** | **1** | **134.09** | **-0.48** | **5** | **1.20** | **1** |
| 4 | Gharounda | 0.55 | 0.60 | 2 | 58.68 | -1.45 | 6 | 104.77 | -1.58 | 6 | -0.25 | 4 |
| 5 | Assandh | 0.53 | -0.13 | 3 | 60.53 | -0.60 | 5 | 175.70 | 1.09 | 1 | 0.02 | 2 |
| 6 | Nilokheri | 0.51 | -0.86 | 5 | 63.59 | 0.81 | 2 | 153.85 | 0.27 | 3 | -0.30 | 5 |
|  | **X=mean** | **0.54** | **0.00** |  | **61.83** | **0.00** |  | **146.72** | **0.00** |  |  |  |
|  | **SD** | **0.03** |  |  | **2.17** |  |  | **26.54** |  |  |  |  |
| **Mewat** | |  |  |  |  |  |  |  |  |  |  |  |
| 1 | Nuh | 0.65 | 0.28 | 4 | 41.88 | 0.31 | 2 | 95.65 | -0.21 | 4 | 0.19 | 2 |
| **2** | **Taoru** | **0.65** | **0.37** | **2** | **48.13** | **1.55** | **1** | **112.68** | **1.22** | **1** | **0.78** | **1** |
| 3 | Ferozpur Zhorka | 0.68 | 0.81 | 1 | 36.51 | -0.77 | 4 | 80.33 | -1.51 | 5 | 0.03 | 4 |
| 4 | Nagina | 0.65 | 0.29 | 3 | 39.56 | -0.16 | 3 | 99.07 | 0.08 | 3 | 0.16 | 3 |
| **5** | **Punhana** | **0.52** | **-1.75** | **5** | **35.67** | **-0.94** | **5** | **103.19** | **0.42** | **2** | **-1.15** | **5** |
|  | **X=mean** | **0.63** | **0.00** |  | **40.35** | **0.00** |  | **98.19** | **0.00** |  |  |  |
|  | **SD** | **0.06** |  |  | **5.00** |  |  | **11.84** |  |  |  |  |

The bold ones are the selected blocks for survey

Source: Table from Singh et al. 2020
