# Supplementary material for: Regional differences in agricultural and socioeconomic factors associated with farmer household dietary diversity in India
Source: PLoS One. 2020 Apr 16;15(4):e0231107. doi: 10.1371/journal.pone.0231107 (PMC7161949; doi:10.1371/journal.pone.0231107)
Supplement: S5 Table — (DOCX) [file pone.0231107.s005.docx]

Table S5. Number of farmer households falling under “Poor”, “Borderline” or “Acceptable” FCS categories*

| **Categorization of Food Consumption Score (FCS)** | | | | | | | |
| --- | --- | --- | --- | --- | --- | --- | --- |
| **State** | **District** | **Poor** | | **Borderline** | | **Acceptable** | |
|  |  | **Number (*n*)** | **%** | **Number (*n*)** | **%** | **Number (*n*)** | **%** |
| Gujarat | Vadodhara | 0 | 0 | 3 | 2 | 190 | 98 |
|  | Bhavnagar | 0 | 0 | 1 | 1 | 176 | 99 |
|  | Banas Kantha | 2 | 1 | 4 | 2 | 177 | 97 |
|  | **Total** | **2** | 0 | **8** | 1 | **543** | 98 |
| Haryana | Karnal | 0 | 0 | 2 | 1 | 184 | 99 |
|  | Bhiwani | 0 | 0 | 4 | 2 | 177 | 98 |
|  | Mewat | 0 | 0 | 2 | 1 | 184 | 99 |
|  | **Total** | **0** | 0 | **8** | 1 | **545** | 99 |
| **Overall** | | **2** | 0 | **16** | 1 | **1088** | 98 |

* FCS categorization by the World Food Program (WFP 2008).
