# Supplementary material for: Regional differences in agricultural and socioeconomic factors associated with farmer household dietary diversity in India
Source: PLoS One. 2020 Apr 16;15(4):e0231107. doi: 10.1371/journal.pone.0231107 (PMC7161949; doi:10.1371/journal.pone.0231107)
Supplement: Attachment — Submitted filename: Untitled document.edited (2).docx [file pone.0231107.s006.docx]

This paper investigated whether there are any regional-level differences in factors associated with farmer household dietary diversity. The paper particularly focused on the dietary diversity of farmer households which has been infrequently studied in India. However, the authors have failed to explain how they define farmer households. Whether their consideration was based on possession of the land or income-earning from farming activities is not clear. What about the tenants – whether they have been included.

This paper has applied the Food Consumption Score (FSC) approach developed by the World Food Programme (2008) to estimate household dietary diversity. The authors have presented a summary of FSC calculated in the paper and discussed its spatial pattern. But it would have been more meaningful if they have classified households as having "poor," "borderline," or "acceptable" food consumption by applying the WFP's recommended cut-offs to the food consumption score. This could provide information on households' food consumption status and which region/state/district is vulnerable. The generated categorical variable could further be used as the response variable in regression analyses, which may give a different result from the present one.

In this paper, primary data collected from two Indian states - Gujarat and Haryana have been analyzed. The authors discussed the sampling strategies adopted in the paper. However, the justification for the selection of the above states was discussed loosely. Both states are relatively small in size but share different geographical and farming characteristics and food habits as well.

The paper used linear regression methods to identify the associations between agricultural (e.g. crop diversity, landholding size, farm income and crops sold) and socioeconomic (e.g. family income, family education, distance to food markets and consumption of domestically produced milk) factors, and FCS. Results presented in Table 3 show drastic variation in parameters across different districts. This variation could be occurred due to change in regions, highlighting region-specific policy focus. But there is a possibility that the variation in parameters could be due to the presence of multicollinearity among some predictors. Landholding size, Per Capita Annual Income, and crops sold to the markets seem to be highly correlated with each other.

The authors have also ignored few important socio-economic variables like caste and region. Such variables are critical in explaining households’ dietary pattern in India. Similarly, policy variables like access to public distribution system have also been ignored. Some variables based on farm policies of these states should have also been considered for better policy implications at regional level.

In summary, the theme discussed in the paper is interesting - particularly in terms of providing useful inputs to farm and health policy. However, the authors have left several scopes for further improvements of the paper. Therefore, the current version of the manuscript could not be approved.
