# Supplementary material for: Regional differences in agricultural and socioeconomic factors associated with farmer household dietary diversity in India
Source: PLoS One. 2020 Apr 16;15(4):e0231107. doi: 10.1371/journal.pone.0231107 (PMC7161949; doi:10.1371/journal.pone.0231107)
Supplement: Attachment — Submitted filename: Response to Reviewers.docx [file pone.0231107.s007.docx]

**Reviewer's Responses to Questions**

**Comments to the Author**

1. Is the manuscript technically sound, and do the data support the conclusions?

The manuscript must describe a technically sound piece of scientific research with data that supports the conclusions. Experiments must have been conducted rigorously, with appropriate controls, replication, and sample sizes. The conclusions must be drawn appropriately based on the data presented.

Reviewer #1: Yes

Reviewer #2: Partly

2. Has the statistical analysis been performed appropriately and rigorously?

Reviewer #1: Yes

Reviewer #2: No

 3. Have the authors made all data underlying the findings in their manuscript fully available?

The [PLOS Data policy](http://www.plosone.org/static/policies.action#sharing) requires authors to make all data underlying the findings described in their manuscript fully available without restriction, with rare exception (please refer to the Data Availability Statement in the manuscript PDF file). The data should be provided as part of the manuscript or its supporting information, or deposited to a public repository. For example, in addition to summary statistics, the data points behind means, medians and variance measures should be available. If there are restrictions on publicly sharing data—e.g. participant privacy or use of data from a third party—those must be specified.

Reviewer #1: Yes

Reviewer #2: Yes

  4. Is the manuscript presented in an intelligible fashion and written in standard English?

PLOS ONE does not copyedit accepted manuscripts, so the language in submitted articles must be clear, correct, and unambiguous. Any typographical or grammatical errors should be corrected at revision, so please note any specific errors here.

Reviewer #1: Yes

Reviewer #2: Yes

 5. Review Comments to the Author

Please use the space provided to explain your answers to the questions above. You may also include additional comments for the author, including concerns about dual publication, research ethics, or publication ethics. (Please upload your review as an attachment if it exceeds 20,000 characters)

Reviewer #1:

The comments on the paper entitled “Regional differences in agricultural and socioeconomic factors associated with farmer household dietary diversity in India” are as follows:
This study tried to understand the regional-level differences in factors associated with farmer household dietary diversity in the states of Haryana and Gujarat in the country. Household dietary diversity is measured by using the Food Consumption Score (FCS). The survey data collected from 1106 households is employed to analyze the objectives of the study. The determinants of household dietary diversity are analyzed through multiple regression models. The model is checked for multi-collinearity by ensuring that VIF values were less than 3. Block fixed effects, as a robustness check, was also included in all the regressions to reduce the effect of individual sites on the results.
The paper is fairly-written, the variables selected for analysis are explained in detail in the methodology section, and the interpretation and discussion of the regression results are fine. The limitations of the study are clearly brought out in the text. I find this paper is fit for publication in the journal. However, author(s) may consider the following minor suggestions:

1. In the light of results of the study, author(s) may suggest some policy measures in the last section for both Haryana and Gujarat. It would enrich the quality of the paper.

Thanks for this great suggestion. We have included policy recommendations that we feel we are able to draw from our study as the concluding sentence (lines 373-75). As different agricultural and socioeconomic factors were associated with FCS in different regions within Gujarat and Haryana, we did not feel comfortable suggesting more specific policy implications for each state based on our results.

“This suggests that future agricultural and socioeconomic policies should consider regional-level variation in the drivers of household dietary diversity by understanding local social and economic contexts.” (Lines 373-75).

2. Please verify the following sentence in the text: all of our results are correlational and based on observation data, and therefore are not causal as mentioned in the last paragraph of page no 15. Study has used regression analysis to understand the determinants, hence the phrase ‘all of our results’ should be replaced with ‘some of our result’.

Thanks for this, I have corrected it (Line 353)

Reviewer #2:

This paper investigated whether there are any regional-level differences in factors associated with farmer household dietary diversity. The paper particularly focused on the dietary diversity of farmer households which has been infrequently studied in India. However, the authors have failed to explain how they define farmer households. Whether their consideration was based on possession of the land or income-earning from farming activities is not clear. What about the tenants – whether they have been included.

Thanks for this great suggestion, I have now described how we defined farmer households in line 118-120.

“A farmer household here refers to a family directly engaged in farming, particularly crop production and not necessarily owning any land. Those who rented or leased land from others were also included. However, those, owning but not cultivating the land themselves were excluded.” (Lines 118-120).

This paper has applied the Food Consumption Score (FSC) approach developed by the World Food Programme (2008) to estimate household dietary diversity. The authors have presented a summary of FSC calculated in the paper and discussed its spatial pattern. But it would have been more meaningful if they have classified households as having "poor," "borderline," or "acceptable" food consumption by applying the WFP's recommended cut-offs to the food consumption score. This could provide information on households' food consumption status and which region/state/district is vulnerable. The generated categorical variable could further be used as the response variable in regression analyses, which may give a different result from the present one.

Thanks for this. It is a very useful suggestion. I have included an additional table (Table S5) showing households falling into each of these categories (poor, borderline, acceptable). I also discussed these results in Section 3.1 (Lines: 233-239). While we appreciate the suggestion of including this variable as a dependent variable, we were unable to do so as there was little variation in this response variable as most farmers (> 97%) were classified as acceptable across all sites (Table S5).

“Further, using the World Food Program’s categorization of FCS scores (WFP 2008), all of the farmer households across all six districts in both states were categorized as “Poor”, “Borderline” and “Acceptable” (Table S5) to see their vulnerability in terms of FCS cutoffs defined by WFP (2008). However, the results suggest that most of the farmer households (98%) fell into the “Acceptable” category. Only 1% of farmer households in one district of Gujarat (e.g. Banas Kantha) fell into the “Poor” category though 1-2% of farmer households across all districts in both states were “Borderline” cases.” (Lines: 233-239).

In this paper, primary data collected from two Indian states - Gujarat and Haryana have been analyzed. The authors discussed the sampling strategies adopted in the paper. However, the justification for the selection of the above states was discussed loosely. Both states are relatively small in size but share different geographical and farming characteristics and food habits as well.

Thanks for your comment. I have now added more detailed justification for selecting Gujarat and Haryana in section 2.1 (Lines: 75-82).

“We selected two Indian states, Gujarat and Haryana (Fig. 1), because these are two states in India where over 70% of rural households depend on agriculture and these two states vary considering crop and income diversity (Saha 2013; MoA 2014a; MoA 2014b; www.esaharyana.gov.in; [www.dag.gujarat.gov.in](http://www.dag.gujarat.gov.in)). For instance, the average crop diversity in Gujarat was 0.73 compared with 0.70 in Haryana. Gujarat, being a large state with a low availability of water for irrigation, could realize cropping intensity of 57% only as compared to 133% in Haryana which is a small state but with plenty of water available for irrigation (Table S1 and S2). Further, these states were also quite divergent in terms of per capita livestock (0.21 v/s 0.36) and poultry (0.31 v/s 1.89%). Thus, these states made an ideal case for a comparative study.” (Lines: 75-82).

The paper used linear regression methods to identify the associations between agricultural (e.g. crop diversity, landholding size, farm income and crops sold) and socioeconomic (e.g. family income, family education, distance to food markets and consumption of domestically produced milk) factors, and FCS. Results presented in Table 3 show drastic variation in parameters across different districts. This variation could be occurred due to change in regions, highlighting region-specific policy focus. But there is a possibility that the variation in parameters could be due to the presence of multicollinearity among some predictors. Landholding size, Per Capita Annual Income, and crops sold to the markets seem to be highly correlated with each other.

Thanks for your comment. We calculated variable inflation factors for each regression and found no evidence of multicollinearity among independent variables we used. Further, to reduce the effect of location, we included block fixed effects in all regressions. I have updated the text in section 2.6 to make these points clearer (lines 194-96).

“We calculated variable inflation factors (VIFs) for each regression and found no evidence of multi-collinearity (VIF < 1.2) among independent variables used. Further, to reduce the effect of location on our results, we included block fixed effects in all regressions.” (Lines: 194-96).

The authors have also ignored few important socio-economic variables like caste and region. Such variables are critical in explaining households’ dietary pattern in India. Similarly, policy variables like access to public distribution system have also been ignored. Some variables based on farm policies of these states should have also been considered for better policy implications at regional level.

Thanks for these comments. Regarding caste and religion (may be not region) we could have included these variables in the analysis if we had collected data related to these variables. Unfortunately, we did not collect such data. In addition, it would be interesting to understand the impacts of site-specific policies and access to the public distribution system. However, our analysis was conducted at the household level and because we included site fixed effects (district, block) this removed any possibility of examining the impact of site-level factors that do not vary across households within a given site. Including site fixed effects allows us to better understand the factors that influence household-level FCS while reducing the impact of variables that are similar across all households in a given site. We, however, agree with the reviewer that future work should examine the impact of such policies of FCS.

In summary, the theme discussed in the paper is interesting - particularly in terms of providing useful inputs to farm and health policy. However, the authors have left several scopes for further improvements of the paper. Therefore, the current version of the manuscript could not be approved.

Thanks for your helpful comments and useful suggestions. We believe that the additions we made have greatly strengthened the paper.

6. PLOS authors have the option to publish the peer review history of their article ([what does this mean?](https://journals.plos.org/plosone/s/editorial-and-peer-review-process#loc-peer-review-history)). If published, this will include your full peer review and any attached files.

If you choose “no”, your identity will remain anonymous but your review may still be made public.

**Do you want your identity to be public for this peer review?** For information about this choice, including consent withdrawal, please see our [Privacy Policy](https://www.plos.org/privacy-policy).

Reviewer #1: No

Reviewer #2: Yes: Amarnath Tripathi

[NOTE: If reviewer comments were submitted as an attachment file, they will be attached to this email and accessible via the submission site. Please log into your account, locate the manuscript record, and check for the action link "View Attachments". If this link does not appear, there are no attachment files to be viewed.]

While revising your submission, please upload your figure files to the Preflight Analysis and Conversion Engine (PACE) digital diagnostic tool, <https://pacev2.apexcovantage.com/>. PACE helps ensure that figures meet PLOS requirements. To use PACE, you must first register as a user. Registration is free. Then, login and navigate to the UPLOAD tab, where you will find detailed instructions on how to use the tool. If you encounter any issues or have any questions when using PACE, please email us at [figures@plos.org](mailto:figures@plos.org). Please note that Supporting Information files do not need this step.
